# Illegal Drugs Laws: Clearing a 50-Year-Old Obstacle to Research

**DOI:** 10.1371/journal.pbio.1002047

**Published:** 2015-01-27

**Authors:** David Nutt

**Affiliations:** Division of Brain Sciences, Imperial College London, London, United Kingdom

## Abstract

The United Nations drug control conventions of 1960 and 1971 and later additions have inadvertently resulted in perhaps the greatest restrictions of medical and life sciences research. These conventions now need to be revised to allow neuroscience to progress unimpeded and to assist in the innovation of treatments for brain disorders. In the meantime, local changes, such as the United Kingdom moving cannabis from Schedule 1 to Schedule 2, should be implemented to allow medical research to develop appropriately.

## Abstract 2

For over 50 years, many medical drugs have been effectively banned from research and clinical treatment in the vain hope that this will stop recreational use. The scientific community should now challenge this pointless ban and get governments to improve their regulations so science can flourish.

## Introduction

Many drugs are made “illegal” in an attempt to reduce their availability and so their harms. This control occurs at both national and international levels—in the latter case, in the United Nations conventions that make a whole range of drugs from cannabis to heroin “illegal.” Many people are aware of the challenges to this system of control in terms of human rights abuses by those who seek to implement a prohibitionist approach to drug control, as well as the failure of, and massive collateral damage from, the “War on Drugs” that is currently being waged to stop drug use (http://www.lse.ac.uk/IDEAS/publications/reports/pdf/LSE-IDEAS-DRUGS-REPORT-FINAL-WEB.pdf). Less well known are the perverse restrictions that these laws have had on pharmacology and therapeutics research. Here I will show how they have led to censoring of life science and medical research, with disastrous consequences that have lasted for more than 50 years and counting.

Recently additional controls have started to be developed, provoked by the fear of so-called “legal highs.” These are drugs that mimic the actions of controlled drugs but are of different chemical structures, so they fall outside the UN conventions or local laws. So, for example, the Republic of Ireland has now banned the sale of any chemical that might be used recreationally, a move that if enforced could stop all pharmaceutical research and development in the country. In the United States, city and state governments often move to outlaw novel drugs before the federal government believes it has sufficient evidence to make that determination. Some have been extreme in their lack of understanding of pharmacology. For example, a bill in Maryland would have outlawed any compound with any binding to any cannabinoid receptor, with no mention of thresholds for binding affinity, whether the ligand had agonist or antagonist efficacy, or whether actions at other receptor sites might moderate overall abuse potential (http://mgaleg.maryland.gov/2013RS/bills/sb/sb0109f.pdf). This demonstrates a very extreme version of prohibition, in which molecular entities that have yet to exist are deemed Schedule 1, as if we had absolute ability to perfectly predict the activity of a novel chemical structure.

## Drug Control Laws

Most national laws controlling “illegal” drugs are based on the UN Single Convention on Narcotic Drugs (1961) and the Convention on Psychotropic Substances (1971) that define a range of substances that are supposedly sufficiently harmful to be removed from the usual sales regulations (see Table 1). They are made “illegal,” which means that punishments are implemented for sale and, in most cases, possession. Some of these can be very severe; e.g., some countries have the death penalty for personal possession of heroin and other opioids [1].

**Table 1:** The current status of drugs in the UN Conventions and UK and US drugs legislation.

**Drug**	**UN Conventions**	**UK Misuse of Drugs Act**	**US Controlled Substances Act**
Amphetamine/ methamphetamine	Schedule II	Schedule 2	Schedule II
Cannabis	Schedules I and IV	Schedule 1 In Sativex = 4	Schedule II
Cocaine	Schedule I	Schedule 2	Schedule II
DMT	Schedule I	Schedule 1	Schedule I
Heroin	Schedule I	Schedule 2	Schedule I
Ketamine	Not listed	Schedule 2	Schedule III
LSD	Schedule I	Schedule 1	Schedule I
MDMA	Schedule I	Schedule 1	Schedule I
Psilocybin	Schedule I	Schedule 1	Schedule I
D9THC (dronabinol)	Schedule II	Schedule 2	Schedule III

The UN and US Schedules use roman numerals, whereas the UK uses Arabic numbers. DMT = dimethyltryptamine. MDMA = methylenedioxymethylamphetamine (ecstasy). LSD = lysergic acid. THC = tetrahydrocannabinol.

However, many “illegal” drugs have medicinal uses: for example, opioids for pain, amphetamines for narcolepsy and attention deficit hyperactivity disorder (ADHD), and even cocaine for local blood control and anaesthesia in ear nose and throat (ENT) surgery. In most Western countries there is an attempt to make the medical use of these exempt from the legal controls that try to limit recreational use. So, in the United Kingdom and US, drugs such as morphine and amphetamine are exempted from the most severe controls that apply to non-medical drugs, such as crack cocaine and crystal meth (see [1]). In practice this means that they are available from pharmacies and most universities can hold them for research purposes.

The problem for researchers comes from two sources: (1) the banning of certain medicines and (2) current regulations limiting the study of the medical potential of drugs, e.g. LSD, psilocybin, and MDMA, that are subject to the most stringent level of control.

## The Banning of Certain Medicines

Many traditional medicines have been defined out of the pharmacopeia by international and national conventions. These include plant sources of DMT such as ayahausca and ibogaine, but the most obvious one is cannabis. Cannabis has been used medically for over 4,000 years [2], yet since the 1961 UN Single Convention on Narcotic Drugs, it has been defined as not having such value. As a result, cannabis is put into Schedule 1. Drugs located in Schedule 1 are subject to the most stringent level of control in most countries in the world (see [1] for a fuller description of these schedules and laws justifying them). This status means that researchers (both preclinical and clinical) require a special licence to hold the drug. In the UK only four (out of many hundred) hospitals have such a licence, though all can hold heroin, a much more harmful and sought-after drug by anyone’s estimate, because heroin is in Schedule 2. These restrictions have meant that research on the medical uses of cannabis has hardly occurred in the past 50 years, despite substantial increase in knowledge of the many pharmacologically active components of the cannabis plant, many of which have medical potential [2]. Moreover, what little research has taken place—such as the development of the cannabis oral spray Sativex—has been delayed by the question of what licence it would be given (now in the UK, it is Schedule 4 despite being identical in pharmaceutical content to plant cannabis, which is still held in Schedule 1].

Similar controls apply in the US, where therapeutic studies on cannabis products have been hampered by intense regulations: in the US only three people hold Drug Enforcement Agency licences to research cannabis clinically. As a result, in many US states the population defied Federal laws and voted for the legalization of medical cannabis (with Colorado and Washington State making recreational use legal as well: http://en.wikipedia.org/wiki/Medical_cannabis_in_the_United_States).

In the UK sub-national democracy for health issues does not exist, so it is estimated that over 30,000 people use medical cannabis illegally, and many get arrested for doing so, particularly as, since 2005, self-medication with cannabis has been specifically excluded as a defence in UK law (despite the fact that it can still be pleaded as a defence for the use of any other “illegal” drug for self-medication) [3].

## How the Law Stops Innovation of New Medicines

Table 2 shows that many popular “illegal” drugs have plausible medical uses. Some of these come from studies that were conducted when they were legal. For instance, LSD was tested in six clinical trials for alcoholism before it was banned in the 1960s. A recent meta-analysis of these studies found an effect-size equal to that of any current treatment for this addiction [4]. So why has the therapeutic potential of LSD not been developed for the past 50 years? The answer is that, because of its Schedule 1 status, research is almost impossible. Most hospitals are banned from holding it, as are many university research institutions. Getting a Schedule 1 licence in the UK takes about a year and costs around £5,000, with £3,000 for the licence and £2,000 for the other requirements such as extra security for the drug cabinets, police checks, etc.

**Table 2:** Demonstrated and potential medical uses of “illegal” drugs.

**Drug**	**Indications *(Potential ones in italics)***	**References**
Cannabis	Pain, spasticity	Abrams DI, et al. (2007) Cannabis in painful HIV-associated sensory neuropathy: a randomized placebo-controlled trial. Neurology 68: 515–521.
		Zajicek JP, et al. (2012) Multiple sclerosis and extract of cannabis: results of the MUSEC trial. J Neurol 83: 1125–1132.
	PTSD	Passie T, Emrich HM, Karst M, Brandt SD, Halpern JH (2012) Mitigation of post-traumatic stress symptoms by *Cannabis* resin: a review of the clinical and neurobiological evidence. Drug Test Anal 4: 649–659.
	Cancer	Stella N, Kline T (2012) Composition and methods of treating glioblastoma. World Intellectual Property Organisation. Publication Number WO 2012/024670 A2.
	ADHD	Strohbeck-Kuehner P, Skopp G, Mattern R (2008) Cannabis improves symptoms of ADHD. Cannabinoids 3: 1–3.
LSD	Addiction	Krebs T, Johansen P-Ø (2012) Lysergic acid diethylamide (LSD) for alcoholism: a meta-analysis of controlled trials. J Psychopharmacol 26: 994–1002.
	Terminal anxiety	Gasser P, Holstein D, Michel Y, et al. (2014) Safety and efficacy of lysergic acid diethylamide-assisted psychotherapy for anxiety associated with life-threatening diseases. Journal of Nervous and Mental Disease. doi: 10.1097/NMD.0000000000000113
MDMA	PTSD	Mithoefer MC, et al. (2013) Durability of improvement in post-traumatic stress disorder symptoms and absence of harmful effects or drug dependency after 3,4-methylenedioxymethamphetamine-assisted psychotherapy: a prospective long-term follow-up study. J Psychopharmacol 27: 28–39.
	*Parkinson disease*	Huot P, et al. (2011) Characterization of 3,4-methylenedioxymethamphetamine (MDMA) enantiomers *in vitro* and in the MPTP-lesioned primate: *R*-MDMA reduces severity of dyskinesia, whereas *S*-MDMA extends duration of ON-time. J Neurosci 31: 7190–7198.
	Brain trauma	Edut S, Rubovitch V, Schreiber S, Pick CG (2011) The intriguing effects of ecstasy (MDMA) on cognitive function in mice subjected to a minimal traumatic brain injury (mTBI) Psychopharmacology 214: 877–889.
Mephedrone	*Cocaine misuse*	Nutt DJ (2011) Perverse effects of the precautionary principle: how banning mephedrone has unexpected implications for pharmaceutical discovery. Adv Psychopharmacol 1: 35–36.
Psilocybin	Cluster headaches	Sewell RA, Halpern JH, Pope HG Jr (2006) Response of cluster headache to psilocybin and LSD. Neurology 66: 1920–1922.
	OCD	Moreno FA, Wiegand CB, Taitano EK, Delgado PL (2006) Safety, tolerability and efficacy of psilocybin in 9 patients with obsessive-compulsive disorder. J Clin Psychiatry 67: 1735–1740.
	*Depression*	Griffiths R, Richards W, Johnson M, McCann U, Jesse R (2008) Mystical-type experiences occasioned by psilocybin mediate the attribution of personal meaning and spiritual significance 14 months later. J Psychopharmacol 22: 621–632.
	Cancer-related depression	Grob CS, Danforth AL, Chopra GS, et al. (2011) Pilot study of psilocybin treatment for anxiety in patients with advanced-stage cancer. Arch Gen Psychiatry 68: 71–78.
	Tobacco addiction	Johnson MW, Albert Garcia-Romeu A, Cosimano MP, Griffiths RR (2014) Pilot study of the 5-HT_2A_R agonist psilocybin in the treatment of tobacco addiction. J Psychopharmacol 28: 983–992.
	Alcoholism	Bogenschutz M, Forcehimes A, Pommy J, Wilcox C, Barbosa P, Strassman R (2014) Psilocybin-assisted treatment for alcohol dependence: A proof-of-concept study. J Psychopharmacol. In press.

Additionally, there is often considerable extra bureaucracy with the need and cost of import licences, since most suppliers are overseas. Moreover, sourcing an LSD formulation for human clinical trial use is close to impossible under current UK and European clinical trial guidelines that require Good Manufacturing Practice (GMP) production compliance (http://eur-lex.europa.eu/legal-content/EN/TXT/PDF/?uri=CELEX:32014R0536&from=EN), as no company we know of in the world is currently approved for this. The situation is somewhat easier in the US and Switzerland, where drugs sourced to high purity, though without the full GMP accreditation, can be used in clinical studies (see [1]). Thus, academic chemistry departments and small chemistry producers can act as providers.

Similar considerations apply to all the drugs in Table 2, although the rising interest in psilocybin as a neuroscience tool and as a possible treatment for obsessive compulsive disorder (OCD) [5] and depression has led to one company developing a GMP supply. However, this does not end the regulatory hurdles as the tableting and dispensing still requires a Schedule licenced site, which are scarce, and as mentioned above, only four hospitals in the UK have a Schedule 1 dispensing licence. Our own experience has shown that overcoming these hurdles—if at all possible—takes several years and increases the cost of this research by about 10-fold over that for “legal” drugs.

The regulations can also be applied arbitrarily to new drugs that are under research. For example, based on the clinical case reports of MDMA helping in the dyskinesia of Parkinson’s disease [6] we began to develop a series of legal MDMA analogues for this indication. In parallel, “head-shops” began selling similar analogues for recreational use. Following some media hysteria, these drugs became banned in the UK, but the legislation was vey broad and so included the compounds we were working on [7]. As a result, this research has now had to stop because not all the various sites on which the work was conducted can afford Schedule 1 licences.

Another recent example is that of ketamine analogues that were being developed as new treatments for pain and depression [1, 8]. Because one or two became available for recreational use (though without any deaths), these and hundreds of other analogues were banned and put in Schedule 1. This effectively stopped research in this field, leaving only ketamine (as it is Schedule 2) available for research. Ketamine is well known to be dependence inducing and to produce significant bladder damage in a proportion of users, so finding safer alternatives was a priority; the fact that all known analogues, including many that may never be developed—let alone tested—are now Schedule 1 drugs means that finding a safer alternative is now almost certainly never going to happen. The pharmaceutical industry is very reluctant to develop drugs that are controlled because of the significant cost implications of the regulatory hurdles and because investors often consider working in the “illegal” drug space to be condoning drug abuse.

One further absurdity of the current approach is that it takes no notice of amount. This means that a single molecule of an “illegal” drug is illegal. This is already limiting PET research with new 5HT2A receptor tracers, where picogramme quantities required for tracer production (well below quantities having psychological effects) need licences [7]. Similarly, research on the epidemiology of new psychoactive substances is limited because once they are made illegal, transferring tiny (sub-active) amounts between research labs becomes subject to complex licence and import–export regulations. In the UK such licences are required for each and every drug separately which massively increases costs. Moreover each are time-limited to only 8 weeks so they need to be renewed repeatedly.

Our work on cannabis has been delayed because it turned out that cannabis placebo is considered a Schedule 1 drug in the UK. This meant that placebo had to be added to our licence and that import and export licences were then required for obtaining it from overseas suppliers. As these licences only last for 8 weeks, they commonly time-expire before the university or the supplier have dealt with the contractual documents. We are currently on our third licence for placebo cannabis and still awaiting supply.

Most researchers do not have the time, money, or energy to work their way through the regulatory jungle. We are the first group in the UK ever to study psilocybin and the first in the 50 years since the regulations were brought in to study LSD. Already the insights gained have transformed our understanding of the role of these drugs and, by inference, the role of 5HT2A receptors in brain function [9], and these findings have now been back-translated into preclinical studies with considerable value [10].

Maybe one could argue that the impairment of research produced by the regulations on “illegal” drugs is worth it because recreational use is reduced. However, it is highly doubtful that this is the case with any of these drugs since they are all readily available from dealers or even over the Internet. Moreover, we can find no instances of diversion of Schedule 1 or Schedule 2 drugs from research labs. So the law simply censors research rather than protects the public; indeed the limitation to clinical research produced by the regulations almost certainly has done much more harm than good to society by impeding medical progress.

## What Is the Solution?

This is remarkably simple; all that needs to happen is for each national government to redefine UN Schedule 1 drugs as Schedule 2 in their country. The governments would still be complying with the UN conventions (i.e., the drugs would still be “illegal”), but the drugs could be held by research establishments and hospitals alongside drugs currently in Schedule 2, e.g., opioids and stimulants. There would be no increased risk of diversion, but a significant easing of the regulatory burden for research. A more rational European approach to GMP production of research compounds for Phase I and II clinical trials would also make clinical research much easier without any significant risk to participants.

As we work towards lifting the ban on pharmacological innovation and research with current Schedule 1 drugs, it will be important to encourage and support the efforts of scientists to oppose harmful new legislation, such as blanket bans on chemical or pharmacological series. In the US, researchers have intervened in these political processes when city-based or state-based proposed legislation has threatened current or upcoming medical research projects (see for example, the testimony to the Maryland Senate provided in the following: http://www.drugpolicy.org/docUploads/Salvia_Packet_02_02_11.pdf).
